# Infectious diseases specialist consultation in *Staphylococcus lugdunensis* bacteremia

**DOI:** 10.1371/journal.pone.0258511

**Published:** 2021-10-12

**Authors:** Erik Forsblom, Emma Högnäs, Jaana Syrjänen, Asko Järvinen

**Affiliations:** 1 Division of Infectious Diseases, Inflammation Center, Helsinki University Hospital and University of Helsinki, Helsinki, Finland; 2 Department of Internal Medicine, Tampere University Hospital, Tampere, Finland; Hong Kong Children’s Hospital, HONG KONG

## Abstract

**Background:**

Commensal coagulase negative *Staphylococcus lugdunensis* may cause severe bacteremia (SLB) and complications. Treatment of SLB is not fully established and we wanted to evaluate if infectious diseases specialist consultation (IDSC) would improve management and prognosis.

**Methods:**

Multicenter retrospective study of SLB patients followed for 1 year. Patients were stratified according to bedside (formal), telephone (informal) or lack of IDSC within 7 days of SLB diagnosis.

**Results:**

Altogether, 104 SLB patients were identified: 24% received formal bedside and 52% informal telephone IDSC whereas 24% were managed without any IDSC. No differences in demographics, underlying conditions or severity of illness were observed between the groups. Patients with bedside IDSC, compared to telephone IDSC or lack of IDSC, had transthoracic echocardiography more often performed (odds ratio [OR] 4.00; 95% confidence interval [CI] 1.31–12.2; p = 0.012) and (OR 16.0; 95% CI, 4.00–63.9; P<0.001). Bedside IDSC was associated with more deep infections diagnosed compared to telephone IDSC (OR, 7.44; 95% CI, 2.58–21.4; p<0.001) or lack of IDSC (OR, 9.56; 95% CI, 2.43–37.7; p = 0.001). The overall mortality was 7%, 10% and 17% at 28 days, 90 days and 1 year, respectively. Considering all prognostic parameters, patients with IDSC, compared to lack of IDSC, had lower 90 days and 1 year mortality (OR, 0.11; 95% CI, 0.02–0.51; p = 0.005) and (OR, 0.22; 95% CI, 0.07–0.67; p = 0.007).

**Conclusion:**

IDSC may improve management and outcome of *Staphylococcus lugdunensis* bacteremia.

## Introduction

*Staphylococcus lugdunensis* is an integral part of the human normal skin microbiota [1]. First identified in 1988, *Staphylococcus lugdunensis* was originally classified as a coagulase-negative staphylococcus (CoNS). However, microbiological elements for identification of *Staphylococcus lugdunensis* are curious. Traditional tube coagulase test, detecting free coagulase, defines the pathogen as coagulase-negative but in screening slide tests it may appear coagulase-positive due to clumping factor production. Furthermore, the capability to bind vitronectin, fibrinogen and extracellular matrix proteins resembles that of coagulase-positive *Staphylococcus aureus* [2, 3]. Reports suggest that the pathogenicity and clinical characteristics of *Staphylococcus lugdunensis* resembles more that of *Staphylococcus aureus* than other CoNS [4]. A recent review on CoNS concluded that *Staphylococcus lugdunensis* is to be regarded as an intermediate between less pathogenic *Staphylococcus epidermidis* and highly pathogenic *Staphylococcus aureus* as it displays clinical features of both groups [5]. In line with this, there are case reports and studies of *Staphylococcus lugdunensis* bacteremia (SLB) causing severe illness e.g. septic shock [6] and infections e.g. spondylodiscitis [7], foreign body infections [8, 9] and endocarditis [10–12]. The overall mortality in reports on SLB has varied from 10 to 45% [11–15].

Despite increasing knowledge that SLB is associated to considerable mortality and that clinical characteristics of SLB resemble that of *Staphylococcus aureus* bacteremia (SAB), few reports on SLB have adopted or incorporated SAB management with well proven positive prognostic impact. Most previous reports on SLB have not included any infectious diseases specialist consultation (IDSC). One report stated that infectious diseases specialists determined whether a case was bacteremia or a contamination of the blood culture and assessed the origin of bacteremia for each case [11]. A second study concluded that all medical records of the patients with SLB were retrieved and systematically reviewed by an infectious diseases specialist [13]. The high mortality would call for actions to improve prognosis in SLB. Major improvements in treatment of SAB have been achieved through IDSC which has led to enhanced diagnostics and eradication of infection foci [16–18], improved choice and duration of antimicrobial therapy [19] and, above all, improved prognosis [20, 21].

The objective here was to investigate how IDSC impact clinical management, disease progression and prognosis during one year follow-up of SLB patients.

## Materials and methods

### Ethics statement

The trial was approved by the institutional review board of Helsinki University Hospital and the head of research at Tampere University Hospital and the ethical committee of Helsinki University Hospital.

### Settings and study population

This was a retrospective multi-center study. Adult patients with at least one blood culture positive for *Staphylococcus lugdunensis* were included from two university hospitals i.e. Helsinki University Hospital and Tampere University Hospital and adjoining central and tertiary (city) hospitals i.e. the Helsinki City Hospitals (Haartman Hospital and Malmi Hospital) and Pirkanmaa City Hospital, Lohja Hospital, Porvoo Hospital, Hyvinkää Hospital, Raasepori Hospital, Kymenlaakso Central Hospital, Etelä-Karjala Central Hospital and Seinäjoki Central Hospital in Finland from January 2002 to December 2018. Central microbiology laboratories analyzed all blood cultures in their area and the recruitment area population was altogether 2.2 million. The only exclusion criterion was age < 18 years. Data collection included sex, age, underlying and co-morbid diseases, illness severity, need for intensive care unit (ICU) treatment, deep infection identification and eradication and administration route and length of antibiotic therapy as well as any IDSC. Infection focus documentation was based on clinical suspicion by the treating physician or verified by radiological, bacteriological, or pathological investigations. Basic laboratory tests, time to defervescence (axillary temperature below 37.5°C) and hospital treatment duration were recorded. Primary endpoint was mortality at 90 days and 1 year. Secondary endpoints were prevalence of deep infection foci and time to defervescence and hospital treatment duration.

### Follow-up time period

Patients were followed retrospectively for up to 1 year. Patients transferred to other hospitals were followed from electronic patient records and direct contact to that hospital.

### Definitions

SLB was defined as nosocomial (healthcare acquired) when the first positive blood culture for *Staphylococcus lugdunensis* was taken ≥ 48 hours after admission to a hospital or with a preceding hospital discharge within 1 week or if the patient was receiving hemodialysis treatment. Community acquired bacteremia occurred when the blood culture was taken ≤ 48 hours of hospital admission. Background diseases were classified according to McCabe´s criteria [22]. Sepsis in combination with hypotension, hypoperfusion, or organ failure was defined as severe sepsis [23]. Endocarditis was categorized according to the modified Duke criteria [24]. Skin and soft-tissue infections as well as deep infection foci including, pneumonia, endocarditis, purulent arthritis, osteomyelitis, deep-seated abscess and any foreign-body infection were recorded. IDSC within seven days of first positive blood culture was documented and classified as: (i) bedside (formal) consultation, (ii) telephone (informal) consultation and (iii) no consultation [25]. Bedside IDSC was defined as a consultation provided by the infectious diseases specialist after direct review of patients including physical examination, patient records review, and recommendations on clinical management and treatment of SLB which were documented into the medical record by the reviewing infectious diseases specialist. The infectious diseases specialist consultant informed the patient of upcoming clinical management and treatment of SLB. Telephone IDSC was defined as a consultation provided by the infectious diseases specialists to the treating physicians through telephone (or other informal) communication, which was then documented into the medical records by the treating physicians. The treating physician informed the patient of upcoming clinical management and treatment of SLB. Detailed description of the content of IDSC are listed in Table 1. Lack of IDSC was defined as no consultation.

**Table 1:** Detailed description of the content of infectious diseases specialist consultation (IDSC) in management of *Staphylococcus aureus* bacteremia (SAB).

Content of infectious diseases specialist consultation in SAB ^A^^,^^B^	
1.	Thorough review of patient medical records.
2.	Thorough physical examination of the patient.
3.	Written directives on follow-up blood cultures.
4.	Written directives on need for radiological examinations.
5.	Written directives on choice and length of antibiotic therapy.
6.	Written directives on removal of infected foreign devices or eradication of possible infection focus.

^**A**^ In bedside IDSC the infectious diseases specialist carries out the consultation according to the content of Table 1.

^**B**^ In telephone IDSC the infectious diseases specialist informs the treating physician by telephone (or other informal communication) on management of SAB according to the content of Table 1.

### Statistical analysis

Data are presented as absolute values and percentages or as means with standard deviations. Categorical variables were compared with Pearson’s X2 -test and non-parametric data was analyzed with Mann–Whitney U-test. Odds ratios (OR) with 95% confidence intervals (CI) were calculated. Univariate factors with p <0.1 were allowed for the Cox proportional regression model (proportional hazards regression). All tests were two-tailed and p <0.05 was considered as significant. Analyses were done using SPSS 12.0 (SPSS Inc., Chicago, IL, USA).

## Results

### Patient characteristics

A total of 104 SLB patients were included. The distribution of SLB cases according to each year and place of diagnosis is presented in S1 Table. Bedside IDSC was received by 25 patients (24%), whereas 54 (52%) were managed through telephone IDSC and 25 (24%) lacked consultation. No differences in gender, age, bacteremia acquisition and underlying conditions were observed between the three groups (Table 2). However, patients who received bedside IDSC had more often undergone a surgery prior to SLB as compared to patients managed without IDSC (p = 0.024) and had more often heart valve prosthesis as compared to patients with telephone IDSC (p = 0.016). Altogether, 2% had severe sepsis and 9% required intensive care unit treatment at time of blood culture collection. However, no differences in severity of illness were seen when comparing bedside IDSC to the two other groups (Table 2).

**Table 2:** Characteristics, predisposing factors and illness severity of 104 patients with *Staphylococcus lugdunensis* bacteraemia stratified according to infectious disease specialist consultation.

Parameters		Bedside consultation^A^	Telephone consultation ^A^	No consultation	Bedside vs. telephone consultation		Bedside vs. no consultation	
N (%)								
		n = 25 (24)	n = 54 (52)	n = 25 (24)	OR (95% CI)	p-value	OR (95% CI)	p-value
**Male gender**	56 (53)	15 (60)	26 (48)	15 (60)	1.62 (0.62–4.23)	0.33	1.00 (0.32–3.10)	1.00
**Age (mean ± SD)**	67 ± 16	66.0 ± 18	66.4 ± 16	70.1 ± 15		0.83		0.54
**Age > 60 years**	74 (71)	18 (72)	37 (69)	19 (76)	1.18 (0.42–3.36)	0.75	0.81 (0.23–2.88)	0.75
**Hospital acquired infection**	46 (44)	11 (44)	25 (46)	10 (40)	0.91 (0.35–2.37)	0.85	1.18 (0.38–3.63)	0.77
**Underlying classification** ^**B**^								
Healthy or nonfatal	34 (33)	12 (48)	15 (28)	7 (28)	2.40 (0.90–6.43)	0.08	2.37 (0.73–7.68)	0.15
Ultimately or rapidly fatal	70 (67)	13 (52)	39 (72)	18 (72)	0.42 (0.16–1.12)	0.08	0.42 (0.13–1.36)	0.15
**Underlying conditions**								
Diabetes and complication	26 (25)	7 (28)	11 (20)	8 (32)	1.52 (0.51–4.55)	0.45	0.83 (0.25–2.78)	0.76
Coronary artery disease	23 (22)	6 (24)	9 (17)	8 (32)	1.58 (0.49–5.06)	0.44	0.67 (0.19–2.33)	0.53
Chronic lung disease	16 (15)	1 (4)	10 (19)	5 (20)	0.18 (0.02–1.52)	0.08	0.17 (0.02–1.55)	0.08
Chronic renal failure ^**C**^	43 (44) ^**1**^	8 (32)	20 (37)	15 (60)	0.89 (0.32–2.49)	0.82	0.34 (0.10–1.14)	0.08
Chronic alcoholism	8 (8)	1 (4)	4 (7)	3 (12)	0.52 (0.06–4.92)	0.56	0.31 (0.03–3.16)	0.30
Corticosteroid therapy^**D**^	7 (7) ^**2**^	1 (4)	4 (7)	2 (8)	0.52 (0.06–4.92)	0.56	0.46 (0.04–5.41)	0.53
Malignancy	31 (30)	5 (20)	19 (35)	7 (28)	0.46 (0.15–1.42)	0.17	0.64 (0.17–2.39)	0.51
**Predisposing factors**								
Injection drug use	2 (2)	1 (4)	1 (2)	0	2.21 (0.13–36.8)	0.57		
Previous surgery ^**E**^	26 (25)	10 (40)	13 (24)	3 (12)	2.10 (0.76–5.80)	0.15	4.89 (1.15–20.8)	0.024
Chronic dialysis	14 (13)	2 (8)	9 (17)	3 (12)	0.44 (0.09–2.18)	0.30	0.64 (0.10–4.19)	0.64
Foreign device^**F**^	44 (42)	12 (48)	21 (39)	11 (44)	1.45 (0.56–3.78)	0.45	1.18 (0.39–3.58)	0.78
Heart valve	5 (5)	4 (16)	1 (2)	0	10.1 (1.07–95.7)	0.016		
Pacemaker	3 (3)	1 (4)	1 (2)	1 (4)	2.21 (0.13–36.8)	0.57	1.00 (0.06–16.9)	1.00
Vascular body	27 (26)	7 (28)	14 (26)	6 (24)	1.11 (0.38–3.22)	0.85	1.23 (0.35–4.37)	0.75
Orthopaedic	16 (15) ^**2**^	4 (16)	8 (15)	4 (16)	1.09 (0.30–4.05)	0.89	0.95 (0.21–4.33)	0.95
**Severity of illness**								
Severe sepsis ^**G**^	2 (2)	0	0	2 (8)				
Intensive care unit^**G**^	9 (9)	2 (8)	4 (7)	3 (12)	1.09 (0.19–6.37)	0.93	0.64 (0.09–4.19)	0.64
Thromboembolic event	7 (7) ^**3**^	1 (4)	4 (7)	2 (8)	0.56 (0.06–5.27)	0.61	0.50 (0.04–5.92)	0.58

Data are number of patients (%) and odds ratios (OR) (95% confidence intervals).

Data

^1^ for 97 patients.

^2^ for 103 patients

^3^ for 100 patients.

^A^ Within 1 week of positive blood culture.

^B^ Underlying diseases according to McCabe and Jackson [22].

^C^ Glomerular filtration rate of <60 mL/min/1.73m2 for more than 3 months.

^D^ Systemic prednisone >10 mg/day or equivalent for over 1 month.

^E^ Surgery 3 months prior to bacteraemia.

^F^ Foreign device inserted prior to bacteraemia.

^G^ At blood culture collection time-point.

### Clinical management and infection foci

Transthoracic echocardiography (TTE) was performed in 80% of patients managed with bedside IDSC whereas only half of those with telephone IDSC (p = 0.012) and to 20% of those with no IDSC (p<0.001) received TTE (Table 3). A similar trend was observed for transesophageal echocardiography (TEE) but there were not statistically significant differences between the groups. Notably, TEE helped to verify suspected infective endocarditis from TTE in 4 patients among the bedside IDSC patients, and no additional infective endocarditis were diagnosed with TEE alone. Similarly, radiological examinations to reveal deep infectious focus, either computer tomography (CT) or magnetic resonance imaging (MRI), were done more often among patients with IDSC as compared to patients managed without any IDSC (Table 3). Altogether, 32% of patients had a deep infection focus and 13% had endocarditis diagnosed. Among patients with bedside IDSC, 68% had a deep infection focus localized and this was significantly more often than among patients managed through telephone IDSC (22%, p<0.001) or without IDSC (16%, p = 0.001). However, no difference in endocarditis or specific deep infection foci subgroups could be revealed between the groups. Surgical infection focus eradication was made to 17% of patients and it was more common in relation to bedside IDSC as compared to telephone IDSC (Table 3). A total of 7 patients were diagnosed with a thromboembolic event. However, among patients with thromboembolic events only 1 patient had endocarditis diagnosed whereas the rest of patients had no diagnosis of endocarditis (Tables 2 and 4).

**Table 3:** Diagnostics, infection foci, antimicrobials and outcome of 104 patients with *Staphylococcus lugdunensis* bacteraemia stratified according to infectious diseases specialist consultation.

Parameters		Bedside consultation ^A^	Telephone consultation ^A^	No consultation	Bedside vs. telephone consultation		Bedside vs. no consultation	
N (%)								
		n = 25 (24)	n = 54 (52)	n = 25 (24)	OR (95% CI)	p-value	OR (95% CI)	p-value
**Radiology** ^**B**^								
Transthoracic echo. ^**C**^	52 (50)	20 (80)	27 (50)	5 (20)	4.00 (1.31–12.2)	0.012	16.0 (4.00–63.9)	< 0.001
Transesophageal echo. ^**C**^	26 (25)	12 (48)	14 (26)	0	2.64 (0.98–7.12)	0.05		
CT or MRI ^**D**^	38 (37)	14 (56)	19 (35)	5 (20)	2.34 (0.89–6.17)	0.08	5.09 (1.45–17.9)	0.009
**Infection foci** ^**B**^								
Any deep infection focus	33 (32) ^**2**^	17 (68)	12 (22)	4 (16)	7.44 (2.58–21.4)	< 0.001	9.56 (2.43–37.7)	0.001
Deep-seated abscesses	3 (3) ^**2**^	3 (12)	0	0				
Foreign body infection	15 (14)	7 (28)	7 (13)	1 (4)	2.61 (0.80–8.50)	0.10	9.33 (1.05–82.8)	0.021
Osteomyelitis	2 (2) ^**2**^	1 (4)	1 (2)	0	2.21 (0.13–36.8)	0.57		
Pneumonia	8 (8) ^**2**^	1 (4)	3 (6)	4 (16)	0.71 (0.07–7.17)	0.77	0.19 (0.02–1.82)	0.12
Endocarditis	14 (13) ^**2**^	6 (24)	7 (13)	1 (4)	2.12 (0.63–7.14)	0.22	6.63 (0.73–60.2)	0.06
Skin infection	19 (18) ^**2**^	5 (20)	9 (17)	5 (20)	1.32 (0.39–4.45)	0.66	0.95 (0.23–3.83)	0.94
**Antibiotic therapy**								
Anti-staphylococcal pen.	43 (41) ^**2**^	16 (64)	24 (44)	3 (12)	2.22 (0.84–5.91)	0.11	11.3 (2.60–48.8)	< 0.001
Any other antimicrobial ^**E**^	57 (55) ^**2**^	9 (36)	30 (56)	18 (72)	0.45 (0.17–1.19)	0.11	0.09 (0.02–0.41)	0.001
Vancomycin	25 (24)	11 (44)	10 (19)	4 (16)	3.46 (1.21–9.84)	0.017	4.13 (1.09–15.6)	0.031
Fluoroquinolone ^**F**^	33 (32) ^**3**^	9 (36)	20 (37)	4 (16)	0.93 (0.35–2.49)	0.88	2.25 (0.57–8.82)	0.24
Rifampicin ^**F**^	17 (16) ^**3**^	11 (44)	5 (9)	1 (4)	7.54 (2.24–25.4)	< 0.001	14.9 (1.72–129)	0.003
**Treatment and outcome**								
Surgical infection removal	18 (17) ^**1**^	9 (36)	5 (9)	4 (16)	5.51 (1.61–18.9)	0.004	2.81 (0.73–10.8)	0.13
Hospitalization, days ± SD	24 ± 23^**4**^	30.1 ± 18	21.8 ± 24	22.3 ± 24		0.01		0.06
Defervescence, days ± S D	2.5 ± 3 ^**5**^	2.70 ± 3	2.91 ± 3	1.20 ± 1.25		0.93		0.05
Defervescense ≤ 7 days	82 (79) ^**5**^	20 (80)	42 (78)	20 (80)	2.38 (0.48–11.9)	0.28	1.00 (0.13–7.81)	1.0
Mortality								
1. Within 28 days	7 (7)	1 (4)	1 (2)	5 (20)	2.21 (0.13–36.8)	0.57	0.17 (0.02–1.55)	0.08
2. Within 90 days	10 (10)	1 (4)	3 (6)	6 (24)	0.71 (0.07–7.17)	0.77	0.13 (0.02–1.19)	0.042
3. Within 1 year	18 (17) ^**1**^	2 (8)	7 (13)	9 (36)	0.61 (0.12–3.18)	0.56	0.16 (0.03–0.85)	0.020

Data are number of patients (%) and odds ratios (OR) (95% confidence intervals).

Data

^1^ for 103 patients.

^2^ for 101 patients.

^3^ for 98 patients.

^4^ for 93 patients.

^5^ for 87 patients.

^A^ Within 1 week of positive blood cultures.

^B^ Radiology and infection foci localized within 3 months.

^C^ Echocardiography.

^D^ Whole-body computed tomography or magnetic resonance imaging.

^E^ II- or III-generation cephalosporin, clindamycin, vancomycin or carbapenems.

^F^ Adjunctive antimicrobial therapy.

**Table 4:** Cox proportional hazards regression model for prognostic factors for 90 days and 1 year mortality of 104 patients with *Staphylococcus lugdunensis* bacteremia (SLB) patients.

**Within 90 days**	**Died**	**Survived**	**OR**	**p-**	**HR**	**p-**
	N = 10 (10)	N = 94 (90)	(95% CI)	**value**	(95% CI)	**value**
Age > 60 years	7 (70)	67 (71)	0.94 (0.23–3.91)	0.93		
Hospital acquired	8 (80)	38 (40)	5.89 (1.19–29.3)	0.017		
Healthy—nonfatal disease ^A^	3 (30)	31 (33)	0.87 (0.21–3.60)	0.85		
Chronic lung disease	4 (40)	12 (13)	4.56 (1.12–18.5)	0.023		
Chronic dialysis	4 (40)	10 (11)	5.60 (1.35–23.3)	0.010		
Intensive care unit^B^	2 (20)	7 (7)	3.11 (0.55–17.5)	0.18		
Mechanical ventilation ^B^	3 (30)	4 (4)	9.64 (1.79–51.9)	0.002	10.4 (1.30–82.8)	0.027
Endocarditis ^C^	2 (20)	12 (13)	1.65 (0.31–8.69)	0.55		
Pneumonia ^C^	2 (20)	6 (6)	3.54 (0.61–20.5)	0.14		
Thromboembolic event ^D^	3 (30)	4 (4)	9.21 (1.71–49.6)	0.003	10.8 (1.58–73.7)	0.015
Any consultation ^E^	4 (40)	75 (80)	0.17 (0.04–0.66)	0.005	0.11 (0.02–0.51)	0.005
**Within 1 year**	**Died**	**Survived**	**OR**	**p-**	**HR**	**p-**
	N = 18 (17)	N = 85 (83)	(95% CI)	**value**	(95% CI)	**value**
Age > 60 years	13 (72)	60 (71)	1.08 (0.35–3.36)	0.89		
Hospital acquired	11 (61)	35 (41)	2.25 (0.79–6.36)	0.12		
Healthy—nonfatal disease ^**A**^	3 (17)	31 (36)	0.35 (0.09–1.29)	0.11		
Chronic lung disease	6 (33)	10 (12)	3.75 (1.15–12.2)	0.022		
Chronic dialysis	5 (28)	9 (11)	3.25 (0.94–11.2)	0.053		
Intensive care unit ^B^	2 (11)	7 (8)	1.39 (0.27–7.33)	0.70		
Mechanical ventilation ^B^	3 (17)	4 (5)	4.29 (0.86–21.2)	0.07		
Endocarditis ^C^	3 (17)	11 (13)	1.40 (0.35–5.68)	0.63		
neumonia ^C^	4 (22)	4 (5)	6.08 (1.35–27.4)	0.010		
Thromboembolic event ^D^	4 (22)	3 (4)	7.43 (1.49–36.8)	0.007	6.80 (1.88–24.6)	0.003
Any consultation ^E^	9 (50)	69 (81)	0.23 (0.08–0.68)	0.005	0.22 (0.07–0.67)	0.007

Data are no. (%) of patients. Hazards ratio (HR) and 95% confidence intervals (95% CI) are presented. NS stands for non-significant.

^A^ Underlying diseases according to McCabe et al. [22].

^B^ At blood culture collection.

^C^ Infection foci within 3 months.

^D^ 2 case of cerebral infarction, 3 cases of pulmonary embolism and 2 cases of deep vein thrombosis.

^E^ Bedside or telephone infectious diseases specialist consultation within 7 days of positive blood cultures.

### Antibiotic susceptibility and therapy

The antimicrobial susceptibility profiles of altogether 102 (98%) patients could be retrieved. Among the available susceptibility data 68% were pan-sensitive and 25% were penicillin-G resistant only and 7% resistant to several different antimicrobials. There were no cases of methicillin resistance. All patients were provided with an antibiotic effective *in vitro* against the *Staphylococcus lugdunensis* strain from the day of the positive blood culture. Two fifths of patients received anti-staphylococcal penicillin whereas 3/5 had other antibiotics (II- or III-generation cephalosporin, clindamycin, vancomycin or a carbapenem). Vancomycin was used as a definitive antibiotic in 24% of patients. Rifampicin and fluoroquinolone were received as adjunctive therapies by 16% and 32% of patients, respectively. Anti-staphylococcal penicillin, vancomycin and rifampicin were provided more often to patients receiving bedside IDSC as compared to patients managed without any IDSC (Table 3). Interestingly, bedside IDSC increased the use of vancomycin and rifampicin as compared to telephone IDSC (Table 3).

### Outcome

Patients receiving bedside IDSC had longer hospital treatment durations as compared to telephone IDSC (p<0.05), whereas no difference in time to defervescense was seen between the consultation groups (Table 3). The overall mortality in the patient population at 28 days, 90 days and 1 year were 7%, 10% and 17%, respectively. No difference in mortality was seen between patients managed through bedside and telephone IDSC. However, patients without IDSC had higher mortality at 90 days and 1 year compared to patients treated with bedside IDSC (p = 0.042 and p = 0.020, respectively) (Table 3).

Factors connected to 90 days and 1 year mortality were analyzed by univariate and Cox regression model (Table 4). In univariate analyses, hospital acquired infection (OR 5.89, p = 0.017), chronic lung diseases (OR 4.56, p = 0.023), chronic dialyses (OR 5.60, p = 0.010), mechanical ventilation at ICU (OR 9.64, p = 0.002) and thromboembolic events (OR 9.21, p = 0.003) were associated to poor outcome but IDSC (OR 0.17, p = 0.005) was linked to better 90 days outcome (Table 4). The same factors, except for hospital acquired infection, were associated in a similar way to 1 year outcome and in addition to them pneumonia (OR 6.00, p = 0.010) was observed as an impairing factor for 1 year survival (Table 4). In Cox regression model, 90 days and 1 year outcome were negatively affected by thromboembolic events (HR 10.8, p = 0.015 and HR 6.80, p = 0.003) and IDSC was an improving factor (HR 0.11, p = 0.005 and HR 0.22, p = 0.007). In addition, mechanical ventilation at ICU impacted 90 days outcome negatively (HR 10.4, p = 0.027) (Table 4).

## Discussion

The main finding of the present study was that SLB patients treated without IDSC had higher mortality at 90 days and 1 year as compared to those treated with bedside IDSC. Furthermore, SLB patients with bedside IDSC, compared to telephone IDSC or lack of IDSC, had a more thorough clinical management including more radiological imaging, more deep infection foci localized and eradicated and received more often narrow spectrum anti-staphylococcal penicillin. Any IDSC was found to be highly important in improving the outcome of SLB patients when all prognostic factors were accounted for in Cox regression model analysis.

Most previous reports on SLB have not provided any IDSC service [12, 14, 15, 26–30] and as far as we know only two reports on SLB have included IDSC to some extent [11, 13]. However, the impact of IDSC on management, disease progression and prognosis of SLB was not evaluated in these reports [11, 13]. Previous reports suggests that the pathogenicity and clinical characteristics of *Staphylococcus lugdunensis* resembles *Staphylococcus aureus* more than that of other CoNS [2–12]. Moreover, the importance of IDSC [16–21] and the superiority of bedside IDSC over telephone IDSC [25] has been observed in SAB. Many of the positive elements of IDSC on clinical management and prognosis observed earlier in studies on SAB were observed in the present study on SLB.

An integral part of clinical management in infectious diseases is the identification of all infectious focuses which necessitates adequate imaging and radiological examinations. The present report observed a strong connection between IDSC and radiological examinations. Among patients with bedside IDSC the vast majority had TTE which was significantly more than patients managed through telephone IDSC or without IDSC. Furthermore, roughly half of patients with bedside IDSC and one fourth of patients with telephone IDSC had TEE performed whereas no TEE were provided to patients who did not receive any IDSC. Whole body CT-scans or MRI were performed significantly more among patients with bedside IDSC as compared to patients receiving no IDSC. Many previous studies on SLB report echocardiography (TTE and TEE) ranging from 23–93% [13, 14, 26–29, 31] but leave CT- or MRI scans undocumented [13, 26, 27, 31] with the exception of two studies reporting 5–9% of patients receiving CT- and/or MRI scan imaging [14, 29]. However, some SLB studies do not report any radiological examinations [11, 12, 15]. The observations of the present study, connecting IDSC to accelerated radiological examinations, is in line with reports on SAB concluding that IDSC enhance radiological investigations [16, 32] and echocardiography (both TTE and TEE) [17, 20, 21, 25, 33].

In the present study, altogether 32% of patients had a deep infection focus identified and 13% had endocarditis diagnosed. These observations are in line with previous reports of various deep infection foci occurring in 0–27% [11, 13, 14, 15, 26, 27, 31, 34] and endocarditis in 8–27% [11, 12, 14, 15, 26, 31, 34] of SLB patients. However, for specific subgroups of infection foci even higher percentages have been reported with one study presenting endocarditis in 46% [13] or foreign body infections in 57% [11] in SLB patients. Notably, one report found no cases of endocarditis in SLB [27]. The present study observed a significant association between IDSC and deep infection focus localization. In SLB patients managed through bedside IDSC roughly 2/3 had a deep infection focus which was significantly more than those receiving telephone IDSC or no IDSC. The observation of IDSC accelerating infection diagnostics is well known from studies on SAB where IDSC, and especially bedside IDSC, have resulted in more localized deep infection foci including endocarditis [16, 17, 25, 32, 33].

Surgical or radiological eradication of infection foci were provided to 17% of patients in the present study. Most reports on SLB do not mention any radiological or surgical eradication of infection focus except for endocarditis [11, 15, 28]. However, for patients with endocarditis studies report cardiac surgery and/or valve replacement procedures for 17–100% of patients [12, 15, 26, 29, 34]. In addition, a case-report presented one SLB patient with endocarditis who underwent cardiac surgery [30]. Previous studies on SAB have concluded that eradication of infection focus is an indispensable part of SAB management and IDSC in SAB is known to accelerate infection foci eradications [18, 19, 32, 35]. We observed a similar trend in the present study where one third of patients with bedside IDSC had infection focus eradication which was significantly more than patients with telephone IDSC.

*Staphylococcus lugdunensis* is susceptible to a wide spectrum of antimicrobial agents–in contrast to other CoNS spices [4]. In the present study, the antimicrobial susceptibility profiles of 99% of the patients could be retrieved of which 68% were pan-sensitive and 25% were penicillin-G resistant only and 7% resistant to several different antimicrobials. Anti-staphylococcal penicillin was received by two fifths of patients whereas three fifths received other antimicrobial agents. Previous studies on SLB report resistance for penicillin or oxacillin varying between 43–85% or 10–42% [11, 12, 26, 29] whereas some authors do not present resistance data for specific antibiotics or antimicrobial subgroups [11]. However, previous SLB studies reporting use of antimicrobials deviate from the present one by use of more broad spectrum antimicrobials: *Choi et al*. and *Fadel et al*. documented 15 and 28 SLB cases (including 20–46% endocarditis) with most patients receiving combination antimicrobial therapies including various broad spectrum antibiotics (e.g. piperacillin-tazobactam or meropenem) whereas only 27–43% patients received monotherapy with a cephalosporin, levofloxacin or vancomycin [12, 27]. In the present study, SLB patients with bedside IDSC had significantly more often anti-staphylococcal penicillin or vancomycin and less often broad spectrum antimicrobial therapy as compared to patients managed without any consultation. Furthermore, adjunctive rifampicin was significantly more common in SLB patients with bedside IDSC as compared to patients receiving telephone IDSC or no consultation. These observations are partly in line with results from studies on SAB. IDSC in SAB is known to improve choice and optimize duration of antimicrobial therapy [16–20, 32, 33] and bedside IDSC is known to accelerate use of adjunctive rifampicin therapy [36].

Mortality in previous reports on SLB has varied. Authors report 30 day mortality of 14% [11], in-hospital mortality of 23% [12] or overall mortality rates of 10–45% [13, 14, 15] whereas two small studies including 6–13 patients have reported no deaths [26, 27]. However, within one subgroup of patients with endocarditis the mortality was reported to be as high as 78% [10]. These mortality rates may be viewed as lower compared those in the present study. We observed that need for mechanical ventilation at the ICU and thromboembolic events were connected to poor 90 days and 1 year mortality in Cox regression model. In contrast, IDSC improved 90 days and 1 year outcome with hazard ratios varying from 0.11–0.22 as compared to no IDSC. Corresponding multivariate analyses of parameters affecting long-term outcome have not been performed in previous reports on SLB patients [11, 12, 15, 26, 27, 29, 31]. However, at least one report has evaluated parameters of all cause in-hospital mortality in SLB patients through univariate analysis presenting Pittsburgh bacteremia score ≥ 2, end-stage renal disease and endocarditis as factors significantly increasing mortality [15]. A probable explanation for the lack of analyses of prognostic parameters in SLB is the scarcity of detailed data on SLB due to the rarity of the disease. The number of patients in many previous reports on SLB has ranged from 6–74 patients [11, 12, 14, 15, 21, 26, 27, 31] with only one report of 100 SLB patients [34]. These SLB patients have been collected throughout time-periods of 1–12 year [11, 12, 14, 15, 26, 27, 29]. The present study included 104 SLB patients over a time-period of 16 years. Hence, as far as we know this is to date the largest SLB study spanning over a longer time-period than earlier reports. Although the total n-number here is larger than in previous reports on SLB, the statistical power of many of the subgroup analyses have suffered due to low n-numbers. We collected data from two large university hospital districts with their own laboratories analyzing all blood cultures from each district. Therefore, we may assume that we have managed to pick up all SLB cases in these areas.

Apart from the small patient number, the retrospective nature of our study is a limitation. First, the present study demonstrated a connection between bedside IDSC and reduced 90 days and 1 year mortality, however, this does not indicate a causal relationship. There is always the possibility that severely ill patients with presumed poor prognosis did not receive bedside IDSC. However, the observation that very few patients deceased during the first 28 days reduced the risk that severely ill patients may have been censured from formal IDSC. Second, the patient cohort was gathered during 2002–2018. Considering the earlier years it is plausible to discuss whether the data are valid for current medical practice. Third, there is currently no consensus on the optimal treatment for SLB. However, regarding SAB it is well known that vancomycin treatment, compared to beta-lactam antibiotics, associates to treatment failures and mortality [37]. In the present study vancomycin therapy was used more often among patients receiving bedside IDSC and it is uncertain if the use of vancomycin for treatment of methicillin-susceptible SLB would have a positive or negative prognostic impact. This is a limitation of the present study. Prospective studies are needed to thoroughly evaluate the potential prognostic impact of various antibiotic therapies in SLB.

In conclusion, we present the largest SLB patient cohort published so far and the results indicate that IDSC, and especially bedside IDSC, may improve clinical management and outcome of SLB patients. These observations suggests that the positive impact of IDSC previously observed in management of SAB may account for management of SLB as well. However, future prospective studies are needed to further evaluate the impact of IDSC in SLB.

## Supporting information

S1 Table 104 *Staphylococcus lugdunensis* bacteraemia patients according to year and place of diagnosis. (DOCX)
