# Supplementary material for: Infectious diseases specialist consultation in Staphylococcus lugdunensis bacteremia
Source: PLoS One. 2021 Oct 12;16(10):e0258511. doi: 10.1371/journal.pone.0258511 (PMC8509883; doi:10.1371/journal.pone.0258511)
Supplement: S1 Table — (DOCX) [file pone.0258511.s001.docx]

S1 Table. 104 *Staphylococcus lugdunensis* bacteraemia patients according to year and place of diagnosis.

| **Year** | **Number of *Staphylococcus lugdunensis* bacteraemia cases** | | |
| --- | --- | --- | --- |
|  | Helsinki and Tampere University Hospital | Adjoining central and tertiary hospitals | In total |
| 2002 | 1 | 0 | 1 |
| 2003 | 3 | 0 | 3 |
| 2004 | 1 | 0 | 1 |
| 2005 | 1 | 0 | 1 |
| 2006 | 0 | 0 | 0 |
| 2007 | 1 | 0 | 1 |
| 2008 | 4 | 0 | 4 |
| 2009 | 3 | 0 | 3 |
| 2010 | 1 | 0 | 1 |
| 2011 | 5 | 0 | 5 |
| 2012 | 3 | 4 | 7 |
| 2013 | 8 | 3 | 11 |
| 2014 | 10 | 2 | 12 |
| 2015 | 10 | 4 | 14 |
| 2016 | 10 | 6 | 16 |
| 2017 | 13 | 7 | 20 |
| 2018 | 2 | 2 | 4 |
|  | 76 | 28 | 104 |
